# HIV-associated synaptic degeneration

**DOI:** 10.1186/s13041-017-0321-z

**Published:** 2017-08-29

**Authors:** Wenjuan Ru, Shao-Jun Tang

**Affiliations:** Department of Neuroscience and Cell Biology, University of Texas Medical Branch, Galveston, TX 77555 USA

## Abstract

Human immunodeficiency virus (HIV) infection induces neuronal injuries, with almost 50% of infected individuals developing HIV-associated neurocognitive disorders (HAND). Although highly activate antiretroviral therapy (HAART) has significantly reduced the incidence of severe dementia, the overall prevalence of HAND remains high. Synaptic degeneration is emerging as one of the most relevant neuropathologies associate with HAND. Previous studies have reported critical roles of viral proteins and inflammatory responses in this pathogenesis. Infected cells, including macrophages, microglia and astrocytes, may release viral proteins and other neurotoxins to stimulate neurons and cause excessive calcium influx, overproduction of free radicals and disruption of neurotransmitter hemostasis. The dysregulation of neural circuits likely leads to synaptic damage and loss. Identification of the specific mechanism of the synaptic degeneration may facilitate the development of effective therapeutic approaches to treat HAND.

## Introduction

There are almost 37 million HIV-infected people worldwide, with over 1 million in U.S in 2015 (https://www.hiv.gov/hiv-basics/overview/data-and-trends/statistics). No cure is currently available. HIV attacks the immune system, especially CD4 T cells, leading to immune dysfunction. Soon after the infection, HIV enters the central nervous system (CNS) and causes neurological dysfunction. Even with the effective anti-retroviral therapy that suppresses viral replication and transmission, about 70% of HIV patients still develop neurological complications [1]. Multiple neurological disorders are manifested in HIV patients.

HIV-associated neurocognitive disorder (HAND) is a common primary neurological disorder associated with HIV infection of the CNS. Patients with HAND often develop cognitive impairment, motor dysfunction and speech problems. Clinical severity of HAND ranges from asymptomatic neurocognitive impairment and mild neurocognitive disorder to HIV-associated dementia (HAD) [2]. Due to the success of HAART, HAD has declined, with a prevalence of less than 5% of HIV patient who are on the treatment [3]. However, the mild forms of HAND are still common and significantly affect a patient’s quality of life.

Neuropathy of the peripheral nerves often develops in HIV patients. With the improved survival of HIV patients on HAART, the prevalence of HIV-associated neuropathy has increased, with about 42% of HIV patients showing neuropathy symptoms [4]. The clinical symptoms include unusual sensation, numbness and severe pain. However, pathological analysis of autopsies indicate that almost all patients with AIDS develop peripheral neuropathy, including those who did not show clinical symptoms [5].

HIV-associated vacuolar myelopathy (VM) is commonly associated with late stages of HIV infection. Of AIDS patients, 20–55% exhibit symptoms of VM [6]. Vacuolization in dorsal and lateral tracts in the thoracic spinal cord is a common pathological characteristic. Patients with VM manifest progressive weakness of legs and sensory abnormalities, and VM may ultimately lead to paralysis of lower limbs [6].

In addition to HIV infection, anti-retroviral therapy may also contribute to neurological disorders. HAART is the current standard treatment for HIV infection. It is a customized combination of different classes of antiretroviral agents, including nucleoside reverse transcriptase inhibitors (NRTIs), non-nucleoside reverse transcriptase inhibitors, protease inhibitors, integrase inhibitors and entry inhibitors. For example, patients treated with NRTIs are prone to develop neuropathy and/or myopathy in a dose-dependent manner [7–9]. A major side effect of protease inhibitors on the CNS is lipodystrophy syndrome, which is characterized by peripheral fat wasting and central adiposity [10]. NRTIs have also been linked to lipodystrophy [11]. HAART was also reported to increase the incidence of encephalitis [12] and induce neuropathy [13].

In this review, we will focus on HAND. In particular, we will critically consider the current understanding of HAND neuropathogenesis from three related aspects: the neuropathogenic underpinnings, the model systems for mechanistic studies, and potential mechanisms of HAND-associated synapse degeneration.

## Neuropathology of HAND

### Early stages

Although 70% of people with HIV have neuropathological abnormalities in the era of HAART [1], only a few studies have reported neuropathology in HIV-infected individuals before the onset of AIDS due to the limited availability of postmortem brains. Most HIV-1 patients remain neurologically unimpaired during early pre-AIDS stages. It generally takes 3 to 6 weeks to become seropositive after HIV infection, and this period is known as seroconversion. During seroconversion, 50–70% of HIV-infected people experience transient “acute HIV syndrome”, such as symptomatic meningitis [14], encephalopathy [15, 16] or myelopathy [17]. Some clinicopathological studies revealed that the CNS entry of HIV-1 might also induce demyelination in the white matter during seroconversion [18, 19].

### Asymptomatic period

After the seroconversion period, HIV infection enters a latency phase called the asymptomatic period, which usually lasts for 8–10 years. Neurological pathologies are noted during this stage, especially in the white matter, although the pathological changes are not consistent. Vascular inflammation is frequently observed in the white matter and basal ganglia, and microglial activation, astrocytosis and myelin pallor are observed in the white matter during this stage [20–22]. Although microglial activation is observed in the cerebral cortex [23], neuronal loss and astrocyte proliferation are rarely seen there [22].

### AIDS stage

Autopsies found that 80–100% of AIDS patients had neuropathological changes in the CNS [24–27]. HIV- associated encephalitis (HIVE) was also observed in some patients at this stage. The neuropathological characteristics of HIVE include microglial nodules, multinucleated giant cells, reactive astrocytosis, microglial proliferation, myelin pallor, and infiltration of peripheral monocytes [28–31]. In contrast to the pre-AIDS stages, when neuronal loss is not seen, neuronal death is frequently observed in AIDS patients [32]. Significant neuronal loss has been reported in the frontal cortex [32–34]. Neuronal death via apoptosis occurs in AIDS patients [35, 36]. Non-apoptotic neuronal injuries, including retraction of dendritic spines, dendritic pruning or aberrant sprouting, axonal disruption and synaptic degeneration, were also observed. Immunostaining analysis of postmortem brain tissues using synaptic and dendritic markers revealed dendritic beading, synaptic degeneration and dendritic spine loss in the brain of HIV patients [37–39]. Axonal injury indicated by elevated neurofilament protein in CSF is also detected in HIV patients [39–41]. Loss of synaptodendritic structures in HIV patients is correlated with reduced volume of neuropil and white matter [41, 42].

## Synaptic degeneration and HAND

Multiple studies have been carried out to identify the neuropathological underpinnings of HAND. Both HIV encephalitis and neuronal loss are observed in the brain after HIV infection, and they appear to associate with severe dementia. However, they do not correlate well with milder forms of cognitive impairment [43]. HIV encephalitis occurs in some but not all HIV-infected individuals. Its presence and severity do not correspond to the degree of cognitive deficits [44–46]. In addition, different from other neurocognitive conditions such as Alzheimer’s or Parkinson’s diseases, the early dementing process in HIV patients is not associated with substantial neuronal apoptosis. Weis et al. reported that AIDS patients with clinical signs of progressive dementia showed no significant difference in neuronal densities compared to patients lacking dementia, indicating that neuronal loss was not causally linked to the development of dementia [33]. Nonetheless, synaptic alteration and degeneration in the brains of HIV patients appear to correlate well with the presence and severity of cognitive impairment [38, 47, 48]. Inhibition of synaptic degeneration may provide an attractive therapeutic target to prevent HAND pathogenesis.

## Animal models of HAND

To investigate the neuropathogenic mechanism of HAND-related pathologies observed in human patients, relevant animal models are essential. Several animal models develop specific aspects of cognitive defects and neuropathological key features of HAND.

### Non-human primate models

The simian immunodeficiency virus (SIV)-infected macaque is an established relevant model for studying the pathogenesis of HAND. In monkeys, SIV can enter the brain shortly after infection and causes brain abnormalities. SIV infection recapitulates the main features of immune response of HIV infection [49–53]. Additionally, HIV-associated neuropathologies in the brains of HIV patients are also developed in the SIV-infected macaque. For example, pre-synaptic damage was reported in SIV-infected macaques, as indicated by elevated levels of neuronal damage marker 14-3-3 protein in the CSF [54, 55]. SIV-infected macaques developed various types of behavioral impairments, similar to those observed in HIV patients, as shown by a number of behavioral and neurophysiological testing modalities [56–60]. This model is particularly useful to study the pathogenesis of HAND in the era of HAART, because the infected macaque can be treated with HAART regimens to mimic the clinical settings [61]. It is also very helpful for the investigation of the synergized effects of drug abuse and HIV infection during neuropathogenesis [62–65]. In addition, because of the multi-time accessibility of CSF, plasma and CNS samples during the progression of infection, this model allows the investigation of the development of HAND through the progressive stages.

Although studies with SIV-infected macaques provide valuable insights into the pathogenesis of HIV infection, it is important to keep in mind that SIV and HIV are not the same. For example, CCR5-preferred HIV can gain the ability to use CXCR4 to enter into monocyte-derived macrophages [66, 67], while CCR5-preferred SIV uses other co-receptors such as CXCR6, GPR15 and GPR but not CXCR4 to enter host cells [68]. To address these limitations, simian-human immunodeficiency virus (SHIV) was constructed, in which the env gene of SIV was replaced by HIV-1 env. Therefore, the hybrid viruses are biologically more similar to HIV than SIV. Macaques infected with SHIV89.6P (CXCR4/CCR5 virus) developed encephalitis characterized by multinucleated giant cells, astrogliosis, microglial nodules, activated macrophages and astrocytes, and perivascular cuffing with mononuclear cells in the white matter [69]. CCR5 (R5)-tropic SHIVSF162P3N virus caused giant cell SIV encephalitis in approximately 30% of infected rhesus macaques that developed AIDS [70]. Giant cell SIV encephalitis lesions included white matter damage, necrosis, and astroglial and microglial activation [70]. SHIVKU, a CXCR4 virus, also could productively replicate in the CNS of rhesus macaques and caused pathological changes [71–73]. Despite the significant contributions of non-human primate models to understanding HIV-1-associated neuropathogenesis, these models are limited by their availability and high cost of maintenance.

### Rodent models

For reasons that are not completely defined, rodents cannot be productively infected by HIV-1. To circumvent this drawback, transgenic mice are generated to express HIV-1 proteins such as the envelope protein gp120 and the transactivator of transcription (Tat), both of which are neurotoxic. In a gp120 transgenic mouse (gp120Tg) model, the gp120 transgene is controlled by the glial fibrillary acidic protein promoter, and thus gp120 is restricted to astrocytes [74]. The release of astrocytically expressed gp120 protein can affect nearby neurons. Confocal imaging of brain sections labeled with dendritic and synaptic markers revealed the dendritic vacuolization, loss of dendritic spines and presynaptic termini in the neocortex and the hippocampus [74]. This gp120Tg mouse also showed reaction of glial cells [74] and impaired proliferation and differentiation of neuronal progenitor cells [75, 76]. Additionally, aging (12 months) gp120Tg mice developed deficits in motor and cognitive performance [74, 77].

In another transgenic mouse model, the Tat transgene is expressed in astrocytes in a Dox-regulated manner [78]. The inducible expression of Tat provides the ability to study the temporal effect of Tat released from astrocytes. This transgenic mouse displays degeneration of neuronal dendrites, neuron death, astrocytosis and enhanced infiltration of activated monocytes and T lymphocytes, and these alterations are largely observed in the cerebellum and cortex [78]. Other studies described more subtle neuronal injuries such as spine loss and synaptic degeneration in hippocampal pyramidal CA1 neurons and striatal neurons [79–81]. The Tat transgenic mice develop impairments in spatial memory and novel object recognition memory [78, 81, 82].

Transgenic mice with full-length [83, 84] or *gag-pol*-deleted HIV-1 genomic DNA [85] have been reported. The integrated HIV-1 genome in the transgenic mouse somewhat resembles HIV-1 provirus. In addition, the transgenic HIV-1 genome has the potential to express multiple HIV-1 proteins. These strengths of this transgenic strategy, however, also complicate the result interpretation for determining the causal relationship between specific HIV-1 proteins and observed phenotypes. Despite low levels of viral protein expression, the full-length transgenic mouse model shows impaired nerve conduction, axonal degeneration and decreased nerve fiber density in the peripheral nervous system. They are also impaired in motor function [83], and show hyper-reaction of microglia and astrocytes [84, 86].

The HIV-1 transgenic rat has been studied by multiple groups as a model of HIV-associated neurological diseases. It contains a *gag*-*pol*-deleted HIV-1 genome that is controlled by the viral promoter. Since without *gag* and *pol* genes that are responsible for viral replication, it cannot produce infectious virions [87]. This rat model expresses multiple viral proteins. In particular, the expression of Tat, gp120, nef and vif RNAs show age-dependent profiles, shifting from peripheral immune organs to the CNS at 10–11 months of age. These features of HIV-1 gene expression indicate that the HIV-1 transgenic rats can model specific aspects of HIV-1-infected individuals on HAART [88]. The 7-to-9-month-old animals show up-regulated expression of neuroinflammation markers such as interleukin-1β (IL-1β), tumor necrosis factor α (TNF-α) and microglial/macrophage marker CD11b [89], which may contribute to the observed synapto-dendritic injury [89]. The transgenic rats develop spatial learning deficits [90, 91] and are impaired in motor performance [92].

The HIV-1 transgenic rodent models described above provide useful tools to study the contribution of viral proteins to the pathogenesis of HAND. However, they have significant limitations. Foremost, they do not acquire HIV-1 infection and thus cannot faithfully model the initial infection stages or the AIDS progression, which are key events associating with HAND development. Understandably, efforts continue to create additional rodent models to mimic HIV infection. One strategy is to introduce human HIV-1 receptors and co-receptors in transgenic rodents [93]. However, it appears that HIV-1 replication was defective in CD4 or CCR5 transgenic rodents [94, 95].

Potash et al. designed a creative approach to generate a novel mouse model of HIV-1 infection. They constructed a chimeric HIV-1 virus by replacing the HIV-gp120 coding region with the gp80 envelope gene from the ectotropic murine leukemia virus. This chimeric virus, called EcoHIV, can enter to the host cells by binding to cationic amino acid transpoter-1 (mCAT) [96]. Despite the widespread expression of mCAT in the mouse tissues, persistent infection seems to be restricted to splenic lymphocytes, peritoneal macrophages and brain [96, 97]. EcoHIV infection by stereotactic inoculation into the mouse basal ganglia caused pre-clinical brain pathology such as microglia and astrocyte activation [96, 98]. However, the lack of gp120 in the chimeric virus presents specific limitations in this model. First, it is unclear to what degree the chimeric virus mimics the HIV-1 infection. For example, it may not target the same populations of cells as HIV-1. In addition, because gp120 is a major HIV-1 neurotoxic protein, this model may not recapitulate some of the neuropathological phenotypes related to HAND.

HIV-infected humanized mice are the exciting new rodent models. One strategy is to generate humanized mice with CNS HIV infection by direct injection of infected human cells. HIV-infected human monocyte-derived macrophages or HIV-infected human microglia cells are injected into the brain of severe combined immunodeficiency deficient (SCID) mice [99, 100] or reconstituted SCID mice with human peripheral blood leukocytes (PBLs) (huPBL/SCID) [101, 102]. SCID and huPBL/SCID mice with the infected human cells recapitulate the several neurological pathologies observed in HIV patients with HIVE, including multinucleated giant cells, astrogliosis, microglial activation and neuronal damage [99–102]. The SCID-HIVE mouse model also develops cognitive deficits. Morris water maze tests revealed their learning and memory impairments, regardless of HAART treatment [103]. Using these models, isolate-specific cognitive deficits and neuropathology were reported. Intracranial injection of macrophages infected with a clade B HIV-1 isolate (HIV-1(ADA)) into SCID mice caused worse performance in cognitive tests and more severe pathological changes than a clade C HIV-1 isolate (HIV-1(Indie-C1)) [104].

Another strategy to generate humanized mice is systemic transplantation of human hematopoietic stem cells (CD34^+^ cells) or adult human peripheral blood mononuclear cells into various immunodeficient mice so that the mice host the human target cells for HIV-1 infection [105–110]. Various neuropathologies were reported in HIV-infected humanized mouse models. For example, NOD/SCID-IL-2Rγ_c_^nul^ (NSG) mice with engrafted human CD34+ stem cells (NSG-hCD34+) developed a functional human immune system containing T lymphocytes, monocytes and macrophages could be efficiently infected with HIV [111–114]. Neuronal and synaptic damages were detected by immunohistochemical staining of various neuronal and synaptic markers such as microtubule associated protein-2, neurofilament and synaptophysin. The neuropathologies appeared to correlate with glial cell activation [112, 113]. The animals also showed memory deficits and persistent anxiety [112, 113]. Although less used for CNS infection, other humanized mouse models (e.g. humanized bone marrow/liver/thymus mouse models) have been used for studies on HIV pathogenesis, transmission, replication and prevention.

### In vitro models

Primary neuron cultures are useful for studying the neurotoxicity of HIV-1 proteins such as gp120 and Tat. Confocal imaging of cultured rat hippocampal neurons revealed that gp120 application caused a dramatic decrease in the number of synapses [115]. Similarly, Tat treatment also induced synaptic loss [116–118]. In addition, gp120 was shown to cause dendritic damage in human primary neurons [115, 119]. Mixed primary cultures that have neurons and glia cells provide an in vitro experimental setting for investigating the interaction between neurons and other cell types (e.g. microglia and astrocytes) during the HIV-induced neuropathogenesis.

## Mechanisms of synaptic degeneration induced by HIV-1 infection

**Fig. 1:**
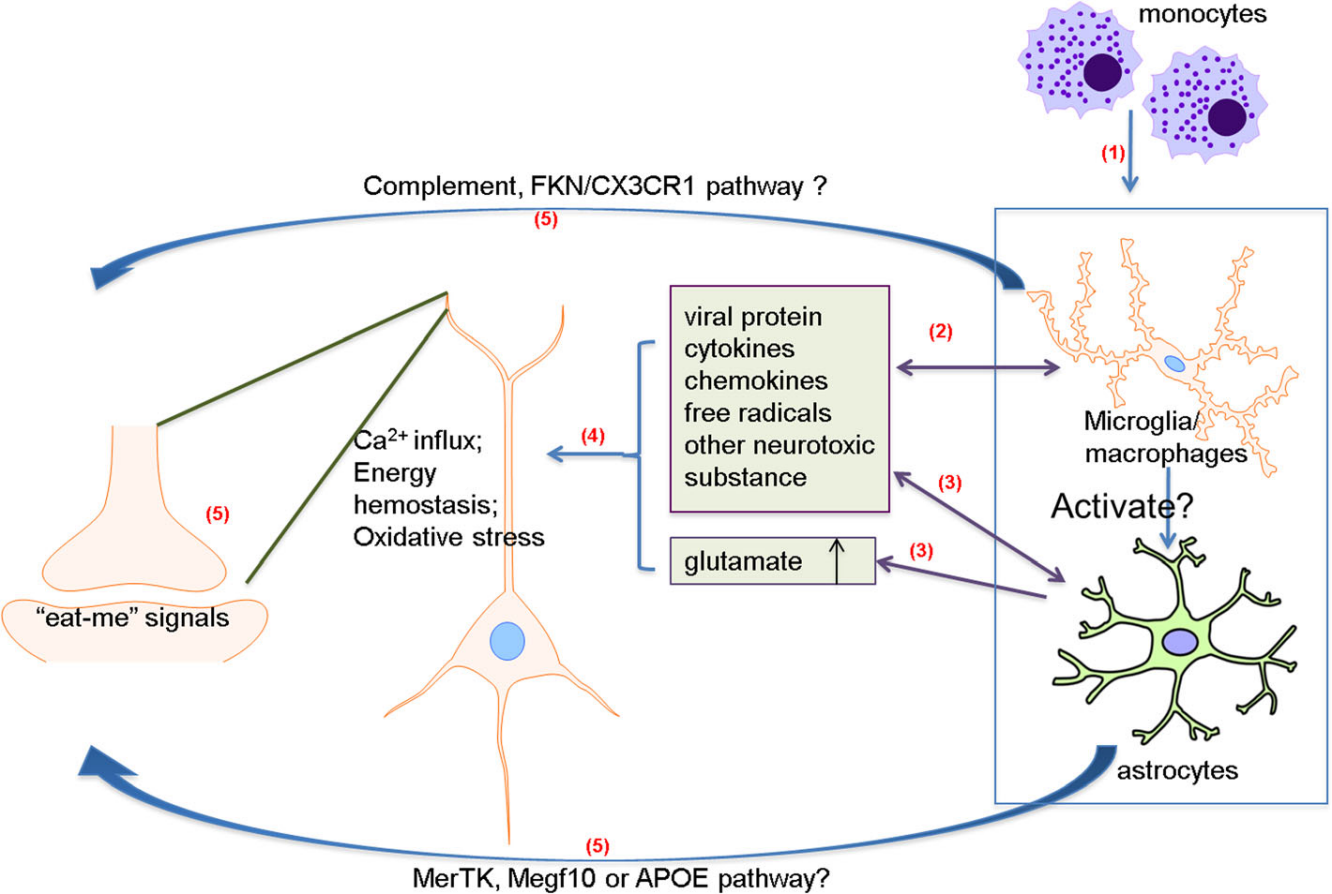
Potential mechanisms of HIV-induced synaptic degeneration. (1) HIV-1 infection of the CNS initiates from transmigration of HIV-1-infected peripheral blood monocytic cells/macrophages across the blood-brain barrier (BBB). Subsequently, microglia and astrocytes become infected and reactivated. (2) The immune-activated and HIV-1-infected microglia/macrophages release viral proteins (e.g. gp120, Tat, Vpr), cytokines (e.g. IL-1β, IL-6, TNF-α), chemokines (e.g. CXCL12, MCP1) and other neurotoxic factors. (3) Infected/reactivated astrocytes can also release neurotoxic substances and pathogenically enhance synaptic activity with increased transmitter release and impaired glutamate re-uptake. (4) The released neurotoxins and extracellular glutamate can cause excessive Ca^2+^ influx, disturbance of energy metabolism and production of reactive oxidative species, which then lead to the disruption of normal neuronal function. On the other hand, the released viral proteins, cytokines, chemokines and free radicals can activate more glial cells and macrophages. (5) These damaged neurons may mark the abnormal synapses with some kind of “eat-me” signals, which can be recognized and eliminated by microglia and/or astrocytes through phagocytotic pathways such as the complementary and FKN/CX3CR1 pathways in microglia or the MerTK, Megf10 and APOE pathway in astrocytes

As HIV-1 cannot infect neurons, HIV-associated synaptic degeneration is likely a bystander effect of the infected cells, including perivascular macrophages, microglia and astrocytes. The infected cells may elicit neurodegenerative responses by releasing viral proteins and other toxic factors such as chemokines and cytokines. The neurotoxins may induce arrays of cellular and molecular cascades that eventually lead to synaptic loss, including Ca^2+^ overload, energy hemostasis disturbance, neurotransmitter (e.g. glutamate) metabolism perturbation, oxidative stresses and excitatory toxicity. In the following sections, we discuss potential mechanisms regulating HIV-induced synaptic degeneration (Fig. 1).

### Neurotoxicity of viral proteins

Viral proteins, particularly gp120 and Tat, are released from infected microglia/macrophages and astrocytes. Gp120 is thought to induce synaptic degeneration via multiple mechanisms. One suggested pathway is glutamate receptor activation-mediated excitotoxicity such as the hyperactivation of N-methyl-D-aspartate receptor (NMDAR) and its associated excessive Ca^2+^ influx [120]. Gp120 can activate NMDARs by binding to their glycine binding sites [121]. Gp120 may also enhance synaptic activity by potentiating the phosphorylation and synaptic trafficking of NMDARs [122]. In addition to stimulating NMDARs, gp120 can bind to its chemokine co-receptor CXCR4 or CCR5 on the neurons to mediate neuronal damage [123, 124]. M-tropic HIV-1 strains preferably bind to CCR5 [125–127], and T-tropic strains use CXCR4 to gain entry into the cells [128]. After binding to its co-receptor, gp120 may facilitate NMDAR activation and intracellular Ca^2+^ increase to induce neuronal damage [129–134] and/or activate signaling cascacades (e.g. ERK and p38 MAPK signaling pathways) that are asssociated with cell damage and death [135–137]. Alternatively, gp120 might cause neurotoxicity via indirect mechanisms. Gp120 can potentiate NMDAR activity by inducing release of proinflammatory cytokines from glial cells [138, 139]. For instance, after binding to the interleukin-1 receptor, IL-1β can stimulate the phosphorylation of NR2B at tyrosine 1472 to potentiate NMDAR activation [138]. In addition, gp120 may cause glial dysfunction and impair extracellular glutamate reuptake. Accumulated extracellular glutamate and NMDAR hyperactivation will induce synaptic damage [140–143]. Furthermore, the neurotoxicity of gp120 may be mediated by down-regulating release of neurotrophic factors (such as BDNF) from activated glia cells [144–148].

By binding to the low-density lipoprotein receptor-related protein (LRP), Tat protein can cause NMDAR activation, excessive Ca^2+^ influx [118, 149–151] and mitochondrial dysfunction [152, 153]. These Tat effects trigger downstream events that contribute to synaptic loss, including the activation of the ubiquitin–proteasome pathway [116, 117], the disturbance of energy metabolism [154] and the production of reactive oxidative species [152, 155]. Tat also stimulates glial cells and macrophages to release cytokines, chemokines and other neurotoxic factors that cause neuronal injury [156–158].

### Neuroinflammation

HIV-1 enter the CNS soon after peripheral infection of blood monocytes and circulating T cells, mainly through a “Trojan horse” mechanism [159] as well as other routes such as “transcytosis” or infection of BBB endothelial cells [160–164]. The viral proteins, inflammatory cytokines and chemokines released from infected and/or activated cells can lead to disruption of BBB integrity and hence exacerbation of the entry of infected cells [165]. As a key component in the BBB structure, astrocytes that become infected can directly cause the increase of BBB permeability [166].

Microglia and perivascular macrophages are CNS-resident immunocompetent cells that can be productively infected by HIV-1. After HIV infection, substantial pro-inflammatory cytokines (e.g. TNF-α, IL-6 and IL-1β) are released from infected/reactivated microglial cells/macrophages [138, 139, 167, 168]. Cytokines in peripheral circulation may also traffic to the CNS [169–171]. The cytokines are elevated in the CSF of HIV patients with cognitive impairments [172, 173]. They may contribute to the pathogenesis of synaptic degeneration via multiple pathways, including NMDAR hyperactivation. For instance, TNF-α and IL-1β can stimulate L-cysteine release from macrophages, which then activates NMDARs to cause neuronal damage [174]. In addition, cytokines may also induce synaptic abnormalities by aberrantly activating cytokine receptors [175–177]. After binding to its receptors on neurons, TNF-α activates multiple pathways that are implicated in neuronal damage, including the nuclear factor-kappa B (NF-*κ*B), ERK, p38 MAPK, the c-Jun N-terminal kinase, and caspase pathways [133, 178].

HIV-infected microglia and macrophages may also release chemokines, which can stimulate neurons via chemokine receptors to induce synaptic degeneration. For example, CXCL12/SDF-1α is elevated in the brain and CSF of HIV patients with HAD [179, 180]. By binding to its receptors, CXCL12 can function as either neuroprotective or neurotoxic mediator [148, 181, 182]. When CXCL12 is cleaved, it switches its preferred receptor from CXCR4 to CXCR3, leading to enhanced neurotoxic effects [183]. Another chemokine, CXCL10, promotes neuron injury by stimulating Ca^2+^ flux [184–186]. Chemokines may also cause neuronal damage by inducing monocyte infiltration. For instance, monocyte chemoattractant protein-1 (MCP1, a.k.a. CCL2) increases in the CSF of HIV patients with cognitive impairment [187], and the MCP-1 increase is implicated in neuronal injury by promoting migration and infiltration of monocytes/macrophages [188–191]. The neuron-released chemokine fractalkine (FKN; a.k.a. CX3CL1), which is also up-regulated in HIV patients [192–195] and has been implicated in HIV-associated dementia [196–198], may also modulate monocyte migration and neuron damage [195, 199–201].

Besides cytokines and chemokines, reactive microglia can also release other neurotoxic substances such as excitatory amino acids, platelet-activating factor and free radicals [202–206]. These neurotoxins may cause NMDAR-mediated excitotoxicity by excessive Ca^2+^ influx and oxidative stress.

Reactive microglia assume diverse phenotypes, which are roughly categorized into the “classical” activation (M1) and “alternative” activation (M2) phenotypes. M1 microglia secrete pro-inflammatory cytokines (e.g. TNF-α, IL-1*β*, interleukin-6 (IL-6)) and reactive oxygen species [207–209], which are implicated in synaptic damage. On the other hand, M2 microglia play a role in repairing neuronal injuries and clearing debris, and they produce anti-inflammatory cytokines and substances such as IL-10, arginase-1 (Arg-1), chitinase 3-like 3 (Chi3l3) and transforming growth factor-β (TGF-β) to facilitate the repair processes [207–209]. Therefore, M1-M2 polarization may play a crucial role in determining the potential neurotoxic or neuroprotective activity of microglia in neurodegeneration disorders [210]. It is currently unknown if dysregulation of M1/M2 polarization of microglia is involved in the pathogenesis of HIV-associated synaptic degeneration.

Although only a small population of astrocytes can be infected by HIV [211–214], the infected astroglia play a critical role in the HIV-associated synaptic injury [213, 215]. Astrocytes are a potentially important reservoir for HIV persistence. In autopsy brain tissues of HIV patients, up to 20% of astrocytes contain integrated HIV-1 [214]. The infected astrocytes produce and secrete viral protein such as gp120, Tat, Vpr, Rev. and Nef, although viral replication is restricted [3, 216–219]. Tat and gp120 can activate astrocytes to produce proinflammatory cytokines such as TNF-α, IL-6 and IL-1β [168, 220], the chemokine CCL5 [221], and neurotoxic nitric oxide (NO) [222], which, as described above, can cause synaptic damage. More recent studies showed that HIV-infected astrocytes could spread the toxic signals to neighboring neurons or un-infected glial cells through gap junctions [223]. The infected astrocytes also increase secretion of CCL2 and glutamate, which may contribute to the dysregulation of the integrity of the BBB as well as defects in monocyte recruitment and immune responses in the CNS [166, 223]. In addition, HIV-infected and/or reactivated astrocytes are probably impaired for glutamate re-uptake, resulting in increased extracellular glutamate and excitotoxicity-induced synaptic degeneration [224–226].

### A role of glia-mediated phagocytosis of synapses?

The discovery of microglial phagocytosis in developmental synaptic pruning [227–229] presents an intriguing possibility of similar mechanisms in synaptic degeneration induced by HIV-1 infection. Microglial phagocytosis is mediated by the classical complement system [229, 230]. More recent work indicates that this microglia-based mechanism is implicated in synaptic loss in Alzheimer’s disease [229, 231] and West Nile virus-induced synaptic loss [232]. Although a role of the complement system was suggested in the immune defense for HIV infection [233, 234], little is known about its involvement in HIV-associated neurodegeneration in the CNS. Complement proteins C1q and C3 are significantly increased in the brains and CSF of HIV patients, and the increase is associated with the up-regulation of the neuronal injury marker neurofilament protein in the CSF and with cognitive impairments [235]. It will be interesting to investigate if complement-mediated microglial phagocytosis contributes to HIV-associated synaptic degeneration. Moreover, the FKN/CX3CR1 pathway also regulates the phagocytosis of microglia [236–238], but its potential contribution to HIV-induced synaptic degeneration has not been tested.

Astrocytes have numerous processes that intimately interact with synapses and monitor synaptic activity. Recent studies indicate that astrocytes can eliminate synapses by phagocytosis [239–241]. Astrocytes express critical regulators of phagocytotic pathways, including Megf10 and MerTK, which play important roles during elimination of synapses in the developing and adult brain [239]. In addtion, the synaptic phagocytic capacity of astrocytes is highly controlled by an APOE isoform in Alzheimer’s disease brains. APOE2 enhances the phagocytic activity of astrocytes; whereas APO4 decreases the rate of synaptic phagocytosis by astrocytes [242]. It is intriguing to conceive that astrocyte dysfunction might contribute to pathogenic synaptic degeneration in the neuropathogenesis of HAND.

## Conclusion

It is clear that HIV-associated synaptic degeneration is a result of cascades of neuropathogenic processes initiated by HIV-1 infection (and often in combination with related comorbidities). The progression of the pathogenesis is determined by the interaction between HIV-1 and the host. The high prevalence of HAND in patients with HAART, which successfully suppresses HIV-1 replication, indicates that intact virions are probably not the major pathogenic agent. Instead, individual HIV-1 toxic proteins such as gp120 and Tat released from infected cells in the CNS may play a major role in inducing the synaptic degeneration. This view posits an interesting and relevant possibility that infected cells that do not productively assemble infective virions, thanks to HAART, may still synthesize pathogenic HIV-1 proteins. The scenario of replication-independent production of HIV-1 protein is superficially counterintuitive, and the underlying mechanism is still poorly understood. Mounting evidence is documenting the neurotoxic effects of individual HIV-1 proteins. Published studies have mainly focused on specific HIV-1 proteins such as gp120 and Tat in different experimental systems, and they have found that more than one HIV-1 protein may elicit complicated molecular pathways that potentially contribute to synaptic degeneration. When these proteins are co-released from the infected cells in the CNS, they likely act in conjunction to cause synaptotoxicity. The conceived interaction of multiple HIV-1 proteins would dramatically increase the complexity of the pathogenic cascades. At the cellular level, in addition to the excitotoxicity from direct stimulation of neurons, reactive microglia and astrocytes likely also attack the neurons at synaptic regions to contribute to the concerted processes of synaptic degeneration. These intrinsically complicating interactions at the molecular and cellular levels in vivo indicate a potential heterogeneity of the pathogenesis among HIV patients, and synaptic degeneration may result from different molecular and cellular pathways elicited by HIV infection in different patients. These conceived complexities and heterogeneity present a daunting task for defining the relevant pathogenic mechanisms in patients for years to come.
